# Editorial: Chemical allergy and the relevance of new models

**DOI:** 10.3389/ftox.2023.1228175

**Published:** 2023-06-14

**Authors:** Marc Pallardy, Valentina Galbiati, Stefan F. Martin

**Affiliations:** ^1^ Inflammation, Microbiome and Immunosurveillance, Inserm, Université Paris-Saclay, Orsay, France; ^2^ Università degli Studi di Milano, Milano, Italy; ^3^ University of Freiburg Medical Center, Freiburg, Germany

**Keywords:** chemical allergy, toxicology, chemical allergens, adverse health effect, immuno response, sensitisation

Chemical allergy represents an adverse health effect that results from an immune response activation following chemical exposure. Among the different forms of chemical allergies, the two most relevant for occupational toxicology are allergic contact dermatitis, and occupational rhinitis and asthma. Chemical allergens are low molecular weight molecules able to activate the immune system. To stimulate an immune response, chemical allergens function as haptens binding to carrier proteins. Several molecules can be involved in chemical allergy either as haptens or immune danger signals such as micro and nano-plastics, particles, pesticides, endocrine disruptors, drugs, fragrances, among others. The initiation and development of chemical allergy phenomena are of considerable interest and importance also for the toxicologist, who has the responsibility of identifying and characterizing the allergenic potential of chemicals and estimating the risk they pose to human health.

This Research Topic aimed to combine interdisciplinary evidence on chemical allergy, covering the biological, pharmaceutical, toxicological, and clinical points of view. The purpose was to evaluate experimental evidence supporting the contribution of these interactions in chemical allergy.

Allergic contact dermatitis is a widespread T cell-mediated inflammatory skin disease, but *in vitro* monitoring of chemical-specific T cells remains challenging. The work proposed by Curato et al., introduced short-term CD154/CD137 upregulation for the detection of human TNBS-specific CD4^+^ and CD8^+^ naive and memory T cells, combining a well-established technique for the generation of TNBS-induced T cell epitopes with recently developed AIM (activation-induced marker) assays for the detection of protein antigen and nickel-specific T cells. Peripheral blood mononuclear cells (PBMC) from healthy donor buffy coats were TNBS-modified and incubated with unmodified PBMC. Activated cells were sorted for re-stimulation and bulk T cell receptor (TCR) high-throughput sequencing (HTS). Among TNBS-specific CD4^+^ T cell clones and lines, 10/13 (77%) responded to TNBS re-stimulation with CD154 upregulation and the presence of different MHC II blocking antibody clones that prevented T cell activation, further confirm antigen-specificity and conventional MHC II restriction for TNBS-specific CD4^+^ T cells. Results were similar for TNBS-specific CD137+CD8^+^ memory T cell clones and lines. In summary, the detection of TNBS-specific T cells by CD154/CD137 upregulation is a fast, comprehensive and quantitative method. Combined with TCR HTS, the mechanisms of chemical allergen recognition that underlie unusually frequent T cell activation can be assessed.

The immunological response in contact hypersensitivity (CHS) is incited by small electrophilic compounds, known as haptens, which react with endogenous proteins after skin absorption. However, the identity of hapten-modified proteins seen as immunogenic remains as yet largely unknown. Adduct measurements have mainly focused on the most abundant blood proteins, human serum albumin (Alb) and hemoglobin (Hb) and in the context of CHS, a large number of mainly *in vitro* studies have been conducted aiming at identifying the most reactive sites of relevant proteins. Despite the extensive mapping of the most reactive sites of relevant proteins, no protein-conjugates have been identified *in vivo* with the exception of the hapten-modified protein in the local lymph nodes of mice treated topically with the model hapten tetramethylrhodamine isothiocyanate (TRITC). The TRITC modification was located on the N-terminal proline of the protein macrophage migration inhibitory factor (MIF). Ndreu et al. focused their attention on the investigation about the presence of this hapten-protein conjugate in blood samples from mice treated topically with TRITC, on TRITC modifications of Alb and Hb, as well as TRITC modifications of MIF other than the N-terminal proline. A proteomic approach was applied to characterize conjugate formation of the aforementioned proteins, using high resolution mass spectrometry (HRMS). No Hb and Alb conjugates were detected. Quantification of both the TRITC-modified and unmodified N-terminal peptide of MIF in blood and lymph node samples gave interesting insights of MIF’s role in murine contact hypersensitivity. Incubation of MIF with four different haptens encompassing different reactivity mechanisms and potencies, showed adduct formation at different amino acid residues, suggesting that MIF can be the preferred target for a wide variety of haptens. The study provides essential progress toward understanding of hapten-protein conjugate formation in CHS and identifies hapten-modified MIF as a potential biomarker for this condition.

Lipids are an important constituent of skin and resulted modified in many skin diseases including psoriasis and atopic dermatitis. The direct effects of common metallic contact allergens on the lipid composition of skin remain an aspect to be better investigated. Knox et al. described the skin lipid profiles in the stratum corneum and viable epidermis of *ex vivo* human skin from a female donor upon exposure to three metal allergens (nickel, cobalt, and chromium). The time-of-flight secondary ion mass spectrometry (ToF-SIMS), which allows for simultaneous visualisation of both the allergen and skin components such as lipids, has been used. Multivariate analysis using partial least squares discriminant analysis (PLS-DA) indicated that the lipid profile of metal-treated skin was different to non-treated skin. Analysis of individual ions led to the discovery that cobalt and chromium induced increases in the content of diacylglycerols (DAG) in stratum corneum. Cobalt also induced increases in cholesterol in both the stratum corneum and viable epidermis, as well as monoacylglycerols (MAG) in the viable epidermis while chromium caused an increase in DAG in viable epidermis in addition to the stratum corneum. In contrast, nickel decreased MAG and DAG levels in viable epidermis. Results obtained in this study indicate that skin lipid content is likely to be altered upon topical exposure to metals, representing a potential implication for the molecular mechanisms by which contact allergens cause skin sensitization.

Since their discovery nearly 40 years ago, B-1 cells have continued to challenge the boundaries between innate and adaptive immunity, as well as myeloid and lymphoid functions. In the Hieronimus and Huaux review, the multifaced role of the B-1 cells is described, highlighting their different roles in both homeostatic and pathological conditions, comprising contact-sensitivity-inducing chemicals. In addition to T cells, also B-1 cells are crucial in the development of contact sensitivity (CS) as supported by the evidence that the transfer of CS-B-1 cells is sufficient to induce CS in untreated mice. After skin sensitization, the hapten-self protein conjugates forming the neo antigens can be drained to the peritoneum, where they encounter and activate B-1 cells. Stimulated B-1 cells migrate to the spleen and lymph nodes via IL-4-related signaling and CXCL13 chemoattraction to produce IgM antibodies specific to the conjugates. This novel IgM circulates in the serum and leads to the recruitment of T cells in lymphoid organs within few hours. Finally, T cells are activated in the spleen by dendritic cells, which are derived from Langerhans cells that have processed neoantigens in the skin. These T cells become CS-effector T cells and return to circulation 4 days after CS initiation. During the second encounter with the chemical, the induced IgM targeting anti-hapten/self-protein conjugate is already circulating and is thus rapidly found in the skin. IgM produced by B-1 cells activates the classical complement pathway and promotes recruitment of T cells, including CS-effector T cells, to the skin for induction of inflammation. Taking into consideration this important evidence about the involvement of B-1 cells in CS, they should not be overlooked when developing new *in vitro* models for predicting the toxicity of chemical compounds.

